# Household possession, use and non-use of treated or untreated mosquito nets in two ecologically diverse regions of Nigeria – Niger Delta and Sahel Savannah

**DOI:** 10.1186/1475-2875-8-30

**Published:** 2009-02-19

**Authors:** Bamgboye M Afolabi, Olayemi T Sofola, Bayo S Fatunmbi, William Komakech, Festus Okoh, Oladele Saliu, Peju Otsemobor, Olusola B Oresanya, Chioma N Amajoh, David Fasiku, Inuwa Jalingo

**Affiliations:** 1 Health, Environment and Development Foundation, P. O. Box 1251, Surulere, Lagos, Nigeria; 2 Federal Ministry of Health, National Malaria and Vector Control Programme, Abuja, Nigeria; 3 Malaria Unit, WHO Nigeria, Abuja, Nigeria; 4 EPI Unit, WHO Nigeria, Abuja, Nigeria; 5 National Population Commission, Abuja, Nigeria

## Abstract

**Background:**

Current use of treated mosquito nets for the prevention of malaria falls short of what is expected in sub-Saharan Africa (SSA), though research within the continent has indicated that the use of these commodities can reduce malaria morbidity by 50% and malaria mortality by 20%. Governments in sub-Sahara Africa are investing substantially in scaling-up treated mosquito net coverage for impact. However, certain significant factors still prevent the use of the treated mosquito nets, even among those who possess them. This survey examines household ownership as well as use and non-use of treated mosquito nets in Sahel Savannah and Niger Delta regions of Nigeria.

**Methodology:**

This survey employed cross-sectional survey to collect data from households on coverage and use of mosquito nets, whether treated or not. Fever episodes in previous two weeks among children under the age of five were also recorded. The study took place in August 1 – 14 2007, just five months after the March distribution of treated mosquito nets, coinciding with the second raining period of the year and a time of high malaria transmission during the wet season. EPI INFO version 2003 was used in data analysis.

**Results:**

The survey covered 439 households with 2,521 persons including 739 under-fives, 585 women in reproductive age and 78 pregnant women in Niger Delta Region and Sahel Savannah Region. Of the 439 HHs, 232 had any mosquito nets. Significantly higher proportion of households in the Niger Delta Region had any treated or untreated mosquito nets than those in the Sahel Savannah Region. In the Niger Delta Region, the proportion of under-fives that had slept under treated nets the night before the survey exceeded those that slept under treated nets in the Sahel Savannah Region. Children under the age of five years in the Niger Delta Region were four times more likely to sleep under treated nets than those in the Sahel Savannah Region.

**Conclusion:**

This study found that despite the fact that treated nets were distributed widely across Nigeria, the use of this commodity was still very low in the Sahel Savannah region. Future campaigns should include more purposeful social and health education on the importance and advantages of the use of treated nets to save lives in the Sahel Savannah region of Nigeria.

## Background

The World Health Organization (WHO) reports that 350–500 million people acquire malaria annually with at least one million of these cases resulting in death [1]. The vast majority of these fatalities occur in sub-Saharan Africa (SSA), and most of the victims are children less than five years of age[2]. In the context of overall childhood mortality, a synthesis of recent studies and reviews suggests that malaria causes at least 20% of all deaths in children under five in Africa [3]. This is astounding for a disease that by and large is preventable, treatable and curable [4].

Long-lasting insecticide-treated mosquito nets (LLIN) are now being promoted as a way to prevent malaria through the distributions of millions of this commodity in Africa and as a tool to gain the attention of the public and raise new funds [4]. Sleeping with the protection of these nets will help prevent the disease [4]. While it is not a total solution, it is a reasonable line of attack in light of today's interdisciplinary approach to combating malaria [4]. Thus, according to WHO, the use of insecticide-treated-nets (ITNs), hereby referred to as treated-mosquito-nets (TMN), is one of the most cost-effective interventions against malaria [5].

High levels of TMN use have been shown to reduce malaria mortality by 17% in children 1–59 months old in African study settings [6] and achieving high levels of TMN use is a key goal of global malaria control efforts by the Roll Back Malaria Partnership [1].

Nevertheless, regardless of all efforts to increase TMN use through the expansion of commercial markets, social marketing interventions, and delivery of this commodity to health facilities and community groups, low level of TMN possession and use characterize most sub-Saharan countries [7-9].

Promotion of mass vaccination presents exclusive opportunity to distribute treated mosquito nets and other child health interventions widely and equitably to the target population of under-5s. Campaigns that integrate measles and polio vaccination, mass treatment of helminthiasis, mass drug administration for lymphatic filariasis, and TMN distribution have been conducted in SSA [6-9]. These campaigns rapidly and equitably increased TMN possession and use [10-14]. Nigeria, like many countries in the African Region, is scaling up delivery of essential health interventions aimed at reducing child and maternal mortality. Integrating service delivery is seen as one option of improving coverage of interventions and the efficiency of health systems. The WHO is supporting Nigeria towards integration of TMN distribution within child survival interventions, especially the Expanded Programme on Immunization (EPI). So far, several rounds of integrated TMN-EPI campaigns have been conducted since April 2006.

A post-campaign household survey was planned to assess, amongst others, the household possession, retention of TMNs and usage by the vulnerable groups during high malaria transmission season, several months after the campaign. The peak malaria transmission in Nigeria falls between June and August, the period when the rains are heaviest. Therefore, the survey was planned to be conducted in August 2007.

The objectives of this study are: (i) to assess and compare the percentage of households in Sahel Savannah and Niger Delta regions of Nigeria with at least one and more than one mosquito net (treated or untreated) (ii) to determine the average number of nets of each type per household (iii) to evaluate household use of treated mosquito nets by visual inspection of mosquito net hanging or not and (iv) to assess and compare the percentage of at-risk groups (under-fives, women in reproductive age and pregnant women) in these areas who slept under a mosquito net (treated or untreated) the night before the survey. Reported here are the results of a household survey to evaluate the use and non-use of treated mosquito nets in two ecologically diverse regions Sahel Savannah in the north and Niger Delta in the south of Nigeria.

## Materials and methods

Although the survey took place in 16 Local Government Areas across the country, these four, Oshimili North and Brass in the southern Nigeria as well as Bungudu and Gulani in the north of the country, were chosen for comparison because of distinct population homogeneity in the north (arid, sudan-Savannah, similar nutritional and living condition) contrasting peculiar population homogeneity in the south (mangrove, riverine, coastal).

### Survey timing and sites

A cross-sectional household survey was performed in August 1 – 14 2007, just five months after the March distribution of TMNs, coinciding with the second raining period of the year and a time of high malaria transmission during the wet season. The survey assessed household possession and use of mosquito nets in the six geo-political zones of the country, assessing one state in each zone and one local government area in each selected state, except for Lagos and Akwa Ibom where two Local Government Areas (LGAs) were surveyed. Using a stratified, two-stage cluster sample design, two districts were selected per region, with probability proportional to estimated population. The distribution of TMNs was planned to be integrated with measles campaign. The objective of this integrated measles-TMN campaign was to make available for each under-five child, one LLIN at the time of measles vaccination campaign. During one week in January 2007, and again in March of the same year, a mass measles campaign was conducted in selected states in Nigeria targeting one million children under the age of five years. Distribution of TMNs was simultaneously on-going, but through the public and private health facilities using different strategies with the same aim of protecting pregnant women and women in the reproductive age group. Between January and March 2007, over one million TMNs were distributed first in 18 states with 36 LGAs and later in 6 states with 44 LGAs. The survey covered 16 LGAs in 14 states within the six geo-political zones. In this study, household possession and use of mosquito nets between two ecologically distinct rural regions in southern and northern Nigeria were compared (Oshimili North and Brass LGA in the Niger Delta (south), population 300,365 as well as Bungudu and Gulani LGAs in the Sahel Savannah (north), population 361,427 including approximately 165,448 under-fives. The immunization campaign, tagged Immunization-Plus Days (IPDs) was proposed by the National Malaria Control Programme of the Federal Ministry of Health in consultation with and supported by in-country partners. The basis for selection included having modest and comparable populations (due to the limited number of TMNs available) and that there was no on-going TMN distribution scheme.

### The study areas

The Niger River enters Nigeria from the north-west, crossing the western part of the country to enter the Atlantic Ocean in the south. Near the coast, the river forms this extensive delta with mangrove forest, lagoons and swamps, stretching over 100 km inland. This area, known as the Niger Delta is the largest in Africa, covering an area of about 36,000 sq. km. Part of this study took place in Brass (latitude 4.19° North and longitude 6.14° East) in Bayelsa State and in Oshimili North in Delta State, with its capital at Asaba, both located in Niger Delta area. Indigenes of Oshimili North speak their own version of Igbo language and are mainly agrarians, though some engage in white-collar jobs. Brass, in Bayelsa State, is the most southerly town in Nigeria located deep into the Atlantic Ocean in the Bight of Biafra. Most families in Brass are of the Ijaw ethnic group, majority of who are fishermen, though some often engage in the production of traditional handcrafts, such as canoes, rope and mats. Malaria transmission in the Niger Delta Area is intense and occurs all year round with peaks after the two rainy seasons (April-July, September-October). The primary vectors in this region are *Anopheles gambiae, Anopheles arabiensis and Anopheles. funestus*.

The Sahel Region of Nigeria serves as a transitional zone between the arid Savannah desert in the north and the wetter tropical areas to the south. Immediately north of the mangrove forest in the south is the Guinea Savannah – a region of tall grasses and trees. Beyond the Guinea Savannah, further north, lies the drier Sudan Savannah, a region of shorter grasses and more scattered, drought-resistant trees such as the baobab, tamarind and acacia. In Nigeria's very dry semi-desert north-eastern corner, a relatively sparse Sahel Savannah vegetation of grasses and shrubs persists. This area also extends to some part of the Northwest. Yobe and Zamfara states, included in this survey, are located in this region. Here, drought and overgrazing have led to desertification. Rainfall averages between 102 and 203 mm per annum and falls mostly from June to September. Most communities are engaged in nomadic herding and limited cultivation of groundnuts and millet. Most of the inhabitants are Muslims.

### Sampling design

Concerning the overall background information for the sample design, the survey was designed to collect data on various variables on ITN indicators in areas where integrated TMN-EPI campaigns were conducted in Nigeria. The major domain to be distinguished in the tabulation of important characteristics was the 48 LGAs, where the programme was implemented in the country. By cluster sampling, 14 out of the 48 LGAs were statistically selected and households within selected LGAs were listed. The population to be covered by the survey was defined as the universe of all. The study primarily focused on the at-risk groups: children age 0–5 years, women in the reproductive age and pregnant women.

### Survey design and sample size

The 2006 census, put the population size of the 48 LGAs at 8,546,280 and the average household size in Nigeria put at five persons (NDHS 2003) translating to about a total of 1,709,256 households in the 48 LGAs.

Therefore, to achieve a 3% precision (level of error) with 95% confidence level assumed proportion of .5 and presumed desire change of 20%, a sample of 1,712 households was required for meaningful analysis (the sample size was adjusted to none response rate of 10%). This translated to 107 households per LGA and 10.7 per cluster. Rounding up the cluster size to 11 households would require drawing 1760 minimum sample size (110 households per selected LGA and 11 per cluster). A multi-stage sample design aimed at selecting 1,760 households from 16 LGAs drawn from 24 states was adopted with equal allocation to all the 16 LGAs (i.e. 110 households per LGA). The first stage was the selection of 16 LGAs from all the project states. The list of all the project states as well as the project LGAs was arranged in zonal order. This arrangement eliminated the concentration of LGAs to be selected in a particular geo-political zone. These 16 LGAs were selected using systematic sample selection procedure. The second stage involved the selection of clusters (localities/Enumeration Area, [EAs]). To ensure that all localities/EAs in the selected LGAs were given equal chance of being selected, the frame of all localities/EAs in the selected LGAs was obtained and 10 clusters systematically selected. The third stage concerned the selection of households from each of the 10 selected clusters. Thrice the number of required households was listed (i.e. 33 households). Then, 11 households systematically selected. It should be noted that the essence of the EA maps was to know and identify the cluster starting point, since from the viewpoint of population, the EAs were not equal in size. The listing continued until the required number (33 households) was obtained.

### Survey

Focus was on children of pre-school age, women in the reproductive age and pregnant women, in line with the priorities of the Ministry of Health, National Malaria Vector and Control Programme. After listing exercise was conducted in survey areas and the houses to be visited were known, experienced field workers (10 from each LGA) were assembled and trained at their locality, in the implementation of the survey questionnaires. Supervisors at Federal and State levels had earlier been trained by Consultants from the WHO and the Nigerian Population Commission. The field workers spent between five to seven days in each survey community, drawing data from 110 households in each of these communities. Data entered into questionnaires were brought back to the WHO Center in Abuja, where recorded data were retrieved and entered into IBM compatible desk top computers using EPI-INFO statistical software.

### Ethical review

The protocol for this study was reviewed and approved by the National Malaria and Vector Control review board. Written or verbal informed consent was received from all participating households.

## Results

This paper compares household possession and use of treated mosquito nets in two diverse ecological areas of Nigeria – the Niger Delta (pop. 300,365) and the Sahel Savannah (pop. 361,427). In all, 220 and 219 households respectively were visited in the Niger Delta Region (NDR) and in the Sahel Savannah Region (SSR). There was no statistical difference (p > 0.5) in whether the respondent was the head of the household or his/her representative. In all, 1,100 (in NDR) and 1,421 (in SSR) persons lived within visited households, giving the average number of persons per household as 5.0 and 6.5 respectively. As shown in Table 1, there were 284 under-fives, 244 women in reproductive age and 28 pregnant women, respectively, in NDR compared with 455, 341 and 50 in SSR respectively. Educational status of women in the reproductive age and socio-economic status of households in NDR were drastically higher than in SSR. Table 2 depicts the distribution of mosquito nets in households in these two regions. In all, only 68 (31.1%) out of 219 households in SSR and 164 (74.5%) out of 220 households in the NDR had mosquito nets of any type. Households in SSR were six times more likely not to possess mosquito nets compared to households in NDR. Households in NDR were more than three times more likely to possess TMNs (108, 49.1%) than their counterpart in SSR (49, 22.3%). In the Niger Delta region, a significant proportion (P = 0.00) of households (108, 49.1%) had treated mosquito nets in contrast to households in SSR (49, 22.3%). Household possession of untreated mosquito nets was significantly higher (P = 0.00) in NDR (56, 25.5%) than in SSR (19, 8.7%). Table 3r explains that out of a total of 164 households with mosquito nets in NDR and 68 in SSR, 134 households (81.7%) and 60 households (88.2%), in NDR and SSR, respectively, had any net hanging (P < 0.001, OR = 4.1), while 89 (40.5%) and 44 (20.1%) had TMNs hanging in these two regions (P < 0.001, OR = 2.7). This implies that the nets were slept under the night prior to the survey. Household possession of any mosquito nets were considerably higher (P < 0.001) in NDR (164, 74.5%) than in SSR (68, 31.1%) and households in NDR are 10 times more likely to possess mosquito nets than in SSR. Table 3 also compares use and non-use of various types of mosquito nets. Mosquito nets seen and reported as hanging suggests that the net was slept under by one or more than one member of the household. A significantly higher proportion (P < 0.001) of households in NDR (164, 74.5%) had mosquito nets in comparison with SSR. When any net possession in the household was disaggregated, HHs in NDR were thrice more likely to possess ordinary mosquito nets than their counterparts in SSR (P < 0.001, OR = 3.0), though no noteworthy difference existed in whether these nets were put to use in the two areas. However, noteworthy difference existed (P < 0.001) in household possession of TMNs in NDR (115, 70.1%) and SSR (49, 22.4%). Households in NDR were almost four times more likely to possess TMNs than households in SSR.

**Table 1:** Characteristics of households (HH) in Sahel Savannah and Niger Delta Areas of survey in Nigeria

	**Area**				
			
	**Niger Delta Region (NDR)**	**Sahel Savannah Region (SSR)**	**χ^2^**	**P value**	**OR (CI)**
Population	300,365	361,427			

Total No. of HH visited	220	219			

Respondent					

Head of HH (%)	137 (62.3)	123 (56.2)			

Representative (%)	83 (37.7)	96 (43.8)			

Total No. persons in HH	1100	1421			

Av. No. of persons per HH	5	6.5			

Total No. of U-5s in HH	284	455			

No. of Women in Reproductive Age (WRA) in HH	244	341			

No. of pregnant women in HH	28	50			

Educational status of WRA in HH					

No formal education	30 (12.3)	265 (77.7)	243.5	0.000	24.9 (15.4; 40.5)

Primary education.	72 (29.5)	48 (14.1)	20.8	0.000	2.7 (1.7; 3.9)

Secondary education.	85 (34.8)	2 (0.6)	131.8	0.000	90.6 (21.6; 539.0)

Post-secondary education.	14 (5.7)	5 (1.5)	8.3	0.004	4.1 (1.35; 13.2)

Socio-economic status (%) (by wealth indices in quintiles)					

Poorest	27.7	41.5			

Moderately poor	20	31.5			

Mildly poor	23.7	17.8			

Poor	16.8	5.5			

Least poor	11.8	3.7			

**Table 2:** Distribution of household possession of treated and untreated mosquito nets in various households of survey in Sahel Savannah and Niger Delta areas of Nigeria.

**Area**					
	**Niger Delta Region (NDR) No. (%)**	**Sahel Savannah Region (SSR) No. (%)**	**χ^2^**	**P**	**OR (CI)**
Total No. of HH	220 (100)	219 (100)			
HH without nets	56 (25.5)	151 (68.9)	83.3	0.000	6.5 (4.2; 10.6)
HH with any nets	164 (74.5)	68 (31.1)			
HH with treated nets	108 (49.1)	49 (22.3)	34.1	0.000	3.4 (2.2; 5.2)
HH with ordinary nets	49 (22.3)	19 (8.7)	21.8	0.000	3.6 (2.0; 6.6)

**Table 3:** Net types and nets seen hanging in households in Sahel Savannah and Niger delta areas of Nigeria

	**Households**				
**Type of mosquito nets and if hanging or not**	**Niger Delta Region (NDR) No. (%)**	**Sahel Savannah Region (SSR) No. (%)**	**χ^2^**	**P**	**OR (CI)**
Any net	164 (74.5)	68 (31.1)	109.8	0.00	10.1 (6.2; 16.5)
Seen hanging (%)	134 (81.7)	60 (88.2)	1.5	0.22	0.6 (0.2; 1.5)
Not hanging (%)	30 (18.3)	8 (11.8)			
					
Ordinary	49 (29.9)	19 (8.7)	15.5	0.00	3.0 (1.7; 5.5)
Seen hanging (%)	45 (91.8)	16 (84.2)	0.9	0.35	2.1 (0.3; 13.1)
Not hanging (%)	4 (8.2)	3 (15.8)			
Treated	115 (70.1)	49 (22.4)	41.9	0.00	3.8 (2.5; 5.9)
Seen hanging (%)	89 (77.4)	44 (89.8)	3.5	0.06	0.4 (0.1; 1.2)
Not hanging (%)	26 (22.6)	5 (10.2)			
**χ^2^**	4.8	0.41			
P	0.028	0.52			
OR	3.29 (1.00;11.87)	0.61 (0.11;3.67)			

Although the distribution campaign increased ITN ownership in households with children aged < 5 years from < 3% to nearly 70% by the end of the campaign, usage of TMN was unexpectedly low since the survey was conducted during the wet season, which has a lot of mosquitoes and high malaria transmission. Nonetheless, usage of TMNs was insignificantly (p = 0.06) higher in the Sahel Savannah area (89.8%) compared to Niger Delta area (77.4%) during the wet season. As shown in Table 4, 305 and 120 mosquito nets were observed in households visited in NDR and in SSR respectively. Average number of nets per HH in NDR was 1.4 compared to 0.55 in SSR. Of the 739 children enumerated in these two ecologically diverse regions, 284 were in NDR and 455 in SSR. Among these, a significant proportion (P < 0.001) in NDR (174, 61.3%) slept under TMNs compared with those in SSR (125, 27.5%). Under-fives in NDR were more than four times likely (OR = 4.2) to sleep under TMNs or more than seven times more likely (OR = 7.5) to sleep under any net than in SSR. Considering usage of mosquito nets by women in reproductive age (WRA), Table 4 also indicates that 244 and 341 of this group were observed in NDR and SSR respectively. Among these, a significant proportion (P < 0.001) in NDR (89, 36.5%) slept under TMNs in NDR when compared with those in SSR (46, 13.5%). WRA were over three times more likely (OR = 3.7) to sleep under TMNs on NDR or more than four times more likely (OR = 4.5) to sleep under any nets than those in SSR. There was no significant difference in the proportion of pregnant women (PW), who slept under TMNs in NDR and SSR though this pattern was altered when any net was considered. In this regard, a noteworthy disparity (P < 0.001) was observed in the proportion of PW who slept under any net in NDR (17, 60.7%) compared SSR (15, 30%).

**Table 4:** Distribution (%) of under-fives (U5s), Women in Reproductive Age (WRAs) and pregnant women (PW) that slept under mosquito nets the night before survey

	**Households**				
	**Niger Delta Region (NDR) No. (%)**	**Sahel Savannah Region (SSR) No. (%)**	**χ^2^**	**P**	**OR (CI)**
Total number of nets	305	120			
Average number of nets per HH	1.4	0.55			
***Children under the age of five years***					
Total No. of under-fives	284	455			
Slept under treated nets	174 (61.3)	125 (27.5)	82.90	0.000	4.2 (3.0; 5.8)
Slept under any nets	225 (79.2)	153 (33.6)	145.51	0.000	7.5 (5.3: 10.8)
Had fever 2 weeks prior to survey	66 (23.2)	129 (28.4)	2.35	0.130	0.8 (0.5:1.1)
***Women in reproductive age (WRA)***					
Total No. of WRA's	244	341			
Slept under treated nets	89 (36.5)	46 (13.5)	42.33	0.000	3.7 (2.4; 5.6)
Slept under any net	121 (49.6)	61 (17.9)	66.69	0.000	4.5 (3.1:6.7)
***Pregnant Women (PW)***					
Total No. of PW	28	50			
Slept under treated net	8 (28.6)	10 (20.0)	0.03	0.850	1.1 (0.3; 3.5)
Slept under any net	17 (60.7)	15 (30.0)	7.00	0.008	3.6 (1.2; 10.7)

**Figure 1:**
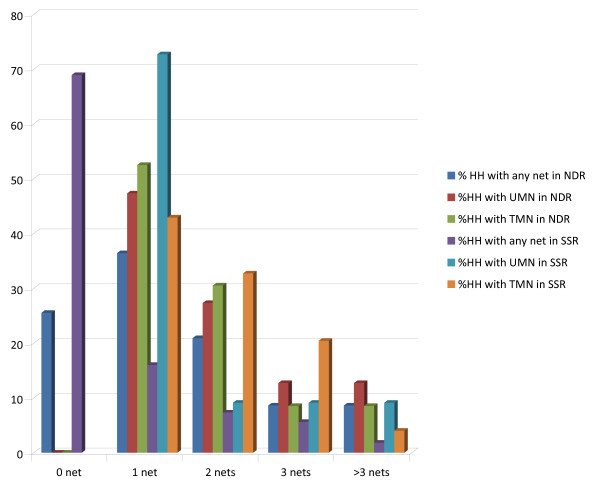
**Percent distribution of mosquito nets by type and by quantity in Niger Delta and Sahel Savannah regions of Nigeria**. (2007).

Table 4 also illustrates that pregnant women in NDR were over three times more likely to sleep under any mosquito nets in NDR than in SSR. Figure 1 shows percent distribution of mosquito nets by type and by quantity in the two regions of consideration. More HHs in the NDR had at least one (36.4%) or two (46, 20.9%), three (19, 8.6%) or more than three any nets compared with HHs SSR that had one (35, 16.0%), two (16, 7.3%), three (12, 5.6%) or more than three (4, 1.8%) any nets. A significant (p < 0.05) proportion of households in NDR (116, 52.3%) had at least one TMN compared to SSR (94, 42.9%). There was no important disparity in household possession of 2 TMNs in both regions. Table 5 shows distribution pattern of ITNs and LLINs during immunization-plus days in various LGAs in Nigeria between 2006 and 2007, indicating widespread delivery of these commodities to various parts of the country during this period.

**Table 5:** Distribution pattern of ITNs* and LLINs** during immunization-plus days in various LGAs in Nigeria between 2006 and 2007

**Year**	**Month**	**Zone**	**State**	**LGA**	**Quantity distributed**	**Campaign**
2006	April	Northwest	Zamfara	Bungudu	30,493*	IPDs
2006	June	Northwest	Zamfara	Anka	11,132*	IPDs
2006	June	Northwest	Jigawa	Malam Madori	12,000*	IPDs
2006	June	Northwest	Kano	Ungogo	4,420*	IPDs
2006	June	Northwest	Kano	Dalla	8,490*	IPDs
2006	September	Northeast	Yobe	Gulani	7,000*	IPDs
2006	September	Northeast	Yobe	Karasuwa	3,300*	IPDs
2006	October	South-south	Cross-River	Abi	10,757**	IMC
2006	October	South-south	Cross-River	Biase	13,044**	IMC
2006	October	South-south	Delta	Oshimili North	10,754**	IMC
2006	October	South-south	Delta	Warri Southwest	6,167**	IMC
2006	October	South-west	Lagos	Epe	10,702**	IMC
2006	October	South-west	Lagos	Ibeju-Lekki	7,795**	IMC
2006	October	South-west	Lagos	Apapa	19,970**	IMC
2006	November	Northeast	Taraba	Gassol	17,000**	IPDs
2007	January	Non-specific 18 States, 36 LGAs		512,645*/**	IPDs	
2007	March	Non-specific 6 States, 44 LGAs		100,000**	IPDs	

LGA = Local Government Areas; IPD = Immunization Plus Days

## Discussion

The United Nations health agency has issued new global guidance on the use of ITNs against malaria, for the first time recommending that they be long-lasting, distributed either free or highly subsidized, and used by all community members to fight a disease that kills more than one million people each year. Until now, the WHO guidelines focused primarily on providing nets for children under five and pregnant women, but recent studies from Kenya show that expanding use of the nets to all people in targeted areas increases coverage and enhances protection of vulnerable groups, while protecting all community members [15]. LLINs were distributed to various communities in Nigeria amongst which were those in Niger Delta states and Sahel Sahara states. Results from our study show that the Immunization-plus days (IPD) campaign, integrating distribution of TMNs with immunization, was successfully carried out in all the six geo-political zones of the country. Measles immunization rates did not appear to be adversely affected by the addition of TMNs into the IPD campaign, and as in Togo, the campaign rapidly increased levels of TMN ownership. Given the most important role that TMNs play in malaria control strategies, one of the primary implications of this campaign was to increase rates of TMN ownership in households and especially among the major at-risk groups including under-fives and pregnant women.

Certain aspects of the Nigerian IPD campaign were unique and deserve particular attention. First, in such a large and populous country with diverse topography ranging from coastal to mangrove, guinea Savannah and Sahel Savannah, this is the first planned integrated campaign covering such an extensive area. Within the first three months of 2006, close to one million TMNs were effectively distributed.

A second aspect unique to immunization-plus days in Nigeria was the one-net-per-mother strategy. This was in contrast to the national campaign in Togo in 2004, which used a one-net-per-child strategy. Because of the large numbers of under-fives in Nigeria, the one-net-per-mother allocation scheme may have been a factor in the campaign's achieving high coverage.

One major finding in this report is the very low educational status of women in the reproductive age in the Sahel Savannah region of Nigeria. This is expected to have influences low usage of TMNs in this region. According to UNICEF, "*Education saves and improves the lives of girls and women. It allows women greater control of their lives and provides them with skills to contribute to their societies. It enables them to make decisions for themselves and influence their families. It is the power that produces all the other developmental and social benefits*" [16]

In this study, there were marked differences in household possession and use of treated mosquito nets between Niger Delta Region and Sahel Savannah Region. This corresponds to the finding in another study in Zambia that reports high possession and low usage of treated mosquito nets [17]. The majority of community members in Sahel Savannah Region are predominantly nomadic cattle rarers. Possibly, in traversing the country, finding grazing grounds for their cattle, nomads in the SSR took their treated mosquito nets with them. This might be the reason why only few households in SSR were observed to have nets in comparison to households in NDR. A study of use of mosquito nets among nomads in Sahel Savannah will clearly answer this dilemma. The Sahel Sahara Region in Nigeria closely shares the same border with Niger Republic where a similar study was conducted in 2005 [18]. Contrary to the findings in Niger Republic, this study found high usage of treated (seen hanging 44/49 or 89.8%) or untreated (seen hanging 16/19 or 84.2%) mosquito nets SSR. In terms of absolute numbers, only 49 out of 110 (22.4%) of households in SSR, compared with 115 (70.1%) in NDR possessed TMNs. Low socio-economic status found in the SSR might have compelled some households to sell the finely-packaged and attractive TMNs to cross-border traders thus depriving themselves of this vital instrument of malaria control. Due to low educational status, households in SSR may not fully appreciate why they have to use the TMNs, or how to unfold, hang and set up these commodities. Some TMNs were reportedly seen within the household, kept as a souvenir.

Although the proportion of mosquito nets seen hanging in SSR was insignificantly higher compared to NDR, few of the at-risk groups, that is, under-fives, women in reproductive age and pregnant women, slept under TMNs the night before the survey. This result varied significantly from Niger Republic [18] study that indicated that 15.4% of under-fives included in their survey reported to have slept under ITNs the preceding night, while in SSR and in NDR, 27.5% and 61.3% of children included in this survey slept under TMNs the preceding night. This was still less than the percentage reported by another study in Zambia [17]. Use of treated mosquito nets by pregnant women is very essential but coverage is still nowhere near the 60% target. According to World Malaria Report [1], Malawi had achieved about 32% coverage of her pregnant women using TMNs, while Nigeria achieved just about 2%. This study however, indicated that 20% of pregnant women in SSR and about 29% in NDR slept under TMN the preceding night and this percentage increased to 30 and 61 respectively when "any net" – treated or untreated was considered. Data from this study also showed 13.5% and 36.5% respectively of women in reproductive age (WRA) sleeping under TMNs the preceding night in SSR and NDR.

Reports from our study showed 52.5% of household in NDR and 42.9% in SSR possessed at least one TMN, which is still slightly lower than data from Zambia [17] that reported 61.3% post-intervention possession in their rural setting. An increased quantity of TMNs in the household to about three or four will most likely bring about herd protection. The effect of increased household possession of TMN means that even though at-risk groups, such as children under the age of five and pregnant women may not sleep under TMNs, as long as lots of these commodities are nearby at night, mosquitoes are likely to be repelled, thus reducing morbidity and mortality due to malaria and saving household money for other essentials of living.

The immunization-plus days campaign delivered key child health interventions (measles vaccine, vitamin A, and treated mosquito nets) to about 80% of children in the target age groups, including a substantial number of children that had not received these interventions before the campaign. Before or during the immunization-plus days campaign, communities to benefit from the distribution of TMNs should receive quality education on proper use of this commodity and its importance to the health and wealth of the households. Beyond this, the Federal Ministry of Health should collaborate very closely with two other vital Ministries – those of Women Affairs and Information – to ensure social mobilization encompasses qualitative education at grass-root. Demonstration of the use of TMNs in the home should be anchored by the Ministry of Information and such demonstrations should take place at market places, town halls and where people mostly gather. Social structure of each community should be taken into consideration as women are not allowed to sit in the same place as men in some communities. In this regards, wide-screen films of use of mosquito nets and dramatization will go a long way in empowering women. Policy makers, traditional heads and religious leader should be mobilized to play vital roles in the distribution and use of TMNs.

A follow-up survey during the rainy season might indicate higher usage rates, as was the case in Togo. In addition, community outreach is advisable to encourage increased TMN usage before the rainy seasons, without any delay.

## Competing interests

The authors have no conflicts of interest concerning the work reported in this paper.

The funders had no role in study design, data collection and analysis, decision to publish, or preparation of the manuscript.

## Authors' contributions

BMA, OTS, BSF and WK conceived the study idea, participated in the study design, were responsible for the micro-planning, overall implementation of the survey including planning, drafting the questionnaire instrument and pre-testing it, and ensuring data management supervision, statistical analysis, report writing and manuscript preparation. They played a vital role in the managerial aspect of the study. FO, SO, PO, OBO and CNA participated in drafting the questionnaire, scheduling of internal responsibilities, cross-checking, field-work, cross-checked the data and took part in statistical analysis. DF and IJ oversaw the research design and methods cross-checked the data and participated in statistical analysis. All authors read and approved the final manuscript.

## References

[B1] World Health Organization World Malaria Report.

[B2] Snow RW, Craig MH, Deichmann U, Marsh K (1999). Estimating Mortality, Morbidity, and Disability Due to Malaria among Africa's Non-pregnant Population. Bulletin of the World Health Organization.

[B3] WHO/UNICEF (2003). The Africa Malaria Report. Geneva.

[B4] Worldwatch, Institute Vital Signs 2007–2008. http://www.WORLDWATCH.ORG.

[B5] Global Malaria Programme Insecticide-treated mosquito nets: A WHO position statement. http://www.un.org/millenniumgoals.

[B6] Lengeler C (2004). Insecticide-treated bed nets and curtains for preventing malaria. Cochrane Database Syst Rev.

[B7] Jones G, Steketee RW, Black RE, Bhutta ZA, Morris SS (2003). How many child deaths can we prevent this year?. Lancet.

[B8] Holtz TH, Marum LH, Mkandala C, Chizani N, Roberts JM, Macheso A, Parise ME, Kachur SP (2002). Insecticide-treated bednet use, anaemia, and malaria parasitaemia in Blantyre District, Malawi. Trop Med Int Health.

[B9] Monasch R, Reinisch A, Steketee RW, Korenromp EL, Alnwick D, Bergevin Y (2004). Child coverage with mosquito nets and malaria treatment from population-based surveys in African countries: a baseline for monitoring progress in Roll Back Malaria. Am J Trop Med Hyg.

[B10] Center for Disease Control (2005). Distribution of insecticide-treated bednets during an integrated nationwide immunization campaign – Togo, West Africa. MMWR Morb Mortal Wkly Rep.

[B11] Center for Disease Control (2006). Distribution of insecticide-treated bednets during a polio immunization campaign – Niger, 2005. MMWR Morb Mortal Wkly Rep.

[B12] Blackburn BG, Eigege A, Gotau H, Gerlong G, Miri E, Hawley WA, Mathieu E, Richards F (2006). Successful integration of insecticide-treated bed net distribution with mass drug administration in Central Nigeria. Am J Trop Med Hyg.

[B13] Grabowsky M, Farrell N, Hawley W, Chimumbwa J, Hoyer S, Wolkon A, Selanikio (2005). Integrating insecticide-treated bednets into a measles vaccination campaign achieves high, rapid and equitable coverage with direct and voucher-based methods. Trop Med Int Health.

[B14] Grabowsky M, Nobiya T, Ahun M, Donna R, Lengor M, Zimmerman D, Ladd H, Hoekstra E, Bello A, Baffoe-Wilmot A, Amofah G (2005). Distributing insecticide-treated bednets during measles vaccination: a low-cost means of achieving high and equitable coverage. Bull World Health Organ.

[B15] World Health Organization WHO releases new guidance on insecticide-treated mosquito nets. http://www.who.int/mediacentre/news/releases/2007/pr43/en/index.html.

[B16] UNICEF (2005). The State of the World's Children 2004.

[B17] Grabowsky Mark, Farrell Nick, Hawley William, Chimumbwa John, Hoyer Stefan, Wolkon Adam, Selanikio Joel (2005). Integrating insecticide-treated bednets into a measles vaccination campaign achieves high, rapid and equitable coverage with direct and voucher-based methods. Trop Med and Int Health.

[B18] Ousmane I, Issifi S, Lama M, Roy J, Hoyer S, Haskew J, Eng J Vanden, Hawley W, Wolkon A, Watkins M, Hochberg N, Eliades M (2006). Distribution of Insecticide-Treated Bednets During a Polio Immunization Campaign Niger, 2005. MMWR Morb Mortal Wkly Rep.

